# Chromophobe renal cell carcinoma: A report of two cases with unusual histological features

**DOI:** 10.4103/0970-1591.38620

**Published:** 2008

**Authors:** Marie Therese Manipadam, Anila Korula, J. Chandrasingh, Antony Devasia

**Affiliations:** Department of General Pathology, Christian Medical College, Vellore, India; 1 Department of Urology, Christian Medical College, Vellore, India

**Keywords:** Chromophobe renal cell carcinoma, eosinophilic variant, ossification

## Abstract

We report two cases of chromophobe renal cell carcinoma with unusual histological features; one case of eosinophilic variant of chromophobe renal cell carcinoma and another case with extensive metaplastic ossification.

Chromophobe renal cell carcinomas (CRCC) comprise about 5% of all renal neoplasms.[1] We present two cases of CRCC with unusual histological features.

## CASE REPORTS

**Figure 1:**
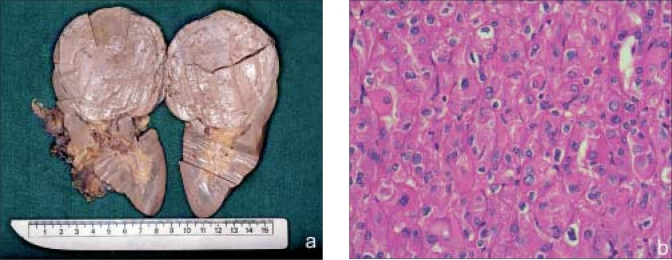
(a) Gross specimen of eosinophilic variant CRCC. Note the tan cut surface; (b) H and E, ×400. The tumor cells with granular eosinophilic cytoplasm and raisinoid nuclei

**Case 1:** A 54-year-old female was incidentally detected to have a large right renal mass while undergoing metastatic workup of a biopsy-proven infiltrating duct carcinoma of the right breast. A CT scan revealed a 6 × 8.1 × 7.3 cm, inhomogenously enhancing lesion in the upper pole of the right kidney. The right radical nephrectomy specimen showed a 7 × 7 × 6 cm tumor at the upper pole with a homogenous, solid light brown cut surface [Figure 1a]. The tumor was limited to the kidney. The H and E stained 4 micron sections showed sheets of polygonal cells with abundant granular eosinophilic cytoplasm with oval nuclei, convoluted nuclear membranes [Figure 1b] and perinuclear cytoplasmic vacuolization. The tumor showed positivity for Cam 5.2, EMA and focally for Vimentin.

**Figure 2:**
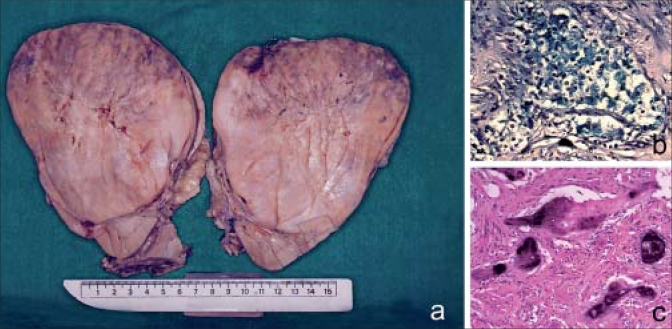
(a) The beige cut surface of the tumor; (b) ×100. Colloidal iron stain with the tumor cells showing diffuse cytoplasmic staining; (c) H and E, ×100. Foci of metaplastic ossification

**Case 2:** A 32-year-old male presented with gross hematuria and loin pain. A CT scan of the abdomen showed a heterogeneously enhancing mass, 10.9 × 12 cm with necrosis and calcifications arising from the upper pole of the right kidney. Radical nephrectomy specimen showed a tumor at the upper pole, 13 × 12 × 13 cm with a beige cut surface and large areas of calcification [Figure 2a]. Microscopy revealed a tumor composed of trabeculae and nests of polygonal cells with distinct cell borders, moderately pleomorphic nuclei with raisinoid appearance, perinuclear halo and abundant pale granular cytoplasm. The tumor cells showed diffuse cytoplasmic reticular staining for colloidal iron [Figure 2b]. There were extensive areas of metaplastic ossification [Figure 2c].

## DISCUSSION

Chromophobe renal cell carcinoma was first reported in humans in 1985. Up to 52% of CRCCs in one series[2] were detected incidentally as in Case 1. Chromophobe renal cell carcinomas are classified into typical and eosinophilic variants depending on the predominant cell type.[3] Three types of cells have been described in CRCCs.[4] The typical CRCCs are composed of cells with thick well-defined borders, wrinkled or raisinoid nuclei and abundant pale granular cytoplasm (the Type III cell) which shows diffuse reticular cytoplasmic staining with Hale's colloidal iron. Eosinophilic variant (EVCRCC) is less frequent and is composed almost completely of Type I cells. The Type I cell is smaller and has granular, eosinophilic cytoplasm. The Type II cell resembles the Type I cell but is larger and has a perinuclear translucent zone. The EVCRCC is not as common as the typical variant and is likely to be mistaken for oncocytoma because of the predominance of Type I cells and hence the significance. The points helping in the differential diagnosis include the sheeting arrangement in EVRCC as opposed to the nested and tubular pattern in oncocytoma, the wrinkled or raisinoid nuclear morphology in EVCRCC as opposed to the round, hyperchromatic nuclei with degenerative atypia in oncocytoma, the well-defined cell borders and the presence of Type II and Type III cells in EVCRCC. Hale's colloidal iron shows a diffuse reticular cytoplasmic staining in CRCC, but oncocytomas may show focal positive staining which is confined to the luminal borders. Electron microscopy is useful for the differential diagnosis in difficult cases, as immunohistochemistry does not help. Ultrastructurally, the oncocytoma cells are packed with mitochondria and the cells in EVCRCC have numerous microvesicles in the cytoplasm.

Chromophobe renal cell carcinomas are quoted as having a better prognosis because these tumors are often localized to the kidneys and are usually of lower Fuhrman's grade. When compared stage for stage, CRCCs have the same prognosis as other RCCs.[5] Tumors with larger size and sarcomatoid change are known to have a worse prognosis.[5,6] Hence, a thorough search for any evidence of sarcomatoid transformation is warranted when a diagnosis of CRCC is made. The eosinophilic variant has been reported to have a better prognosis than typical CRCC in one study.[5]

The interesting feature in Case 2 is the extensive ossification, evident grossly and microscopically. Although calcification is known to occur in RCCs, reports of extensive ossification in CRCC are rare, with only two reports to date.[7,8] The ossification was present in the fibrous stroma in all the cases. The histogenesis is considered to be from stromal osseous metaplasia.

In summary we report two cases of CRCCs with unusual histological features. EVCRCC is a close differential for oncocytoma.

## References

[1] Murphy WM, Grignon DJ, Perlman EJ (2004). Kidney tumours in adults. Tumors of the Kidney, Bladder and related Urinary structures. AFIP atlas of tumour pathology. Series 4.

[2] Crotty TB, Farrow GM, Weiber MM (1995). Chromophobe RCC: Clinicopathological features of 50 cases. J Urol.

[3] Bruce Latham, Dickersin GR, Oliva E (1999). Subtypes of chromophobe RCC: An ultrastructural and histochemical study of 13 cases. Am J Surg Pathol.

[4] Akhthar M, Kardar H, Linjawi T, McClintock J, Ali MA (1995). Chromophobe cell carcinoma of the kidney: A clinicopathologic study of 21 cases. Am J Surg Pathol.

[5] Onishi T, Oishi Y, Yauda S, Abe K, Hasegawa T, Maeda S (2002). Prognostic implications of histological features in patients with chromophobe RCC. BJU Int.

[6] Renshaw A, Henske EP, Loughlin KR, Shapiro C, Weinberg DS (1996). Aggressive variants of chromophobe RCC. Cancer.

[7] Yokozaki H, Ukai R, Kawashita E, Ikeda H, Kuniyasu H, Tahara E (2000). Chromophobe renal cell carcinoma with osseous metaplasia: a case report. Jpn J Clin Oncol.

[8] Wu SL, Fihrmann IJ, Shannon RL (2002). Chromophobe carcinoma with extensive calcification and ossification. Ann Diagn Pathol.

